# *Helicobacter pylori* infection in HIV-positive patients from the Mureş regional center

**DOI:** 10.1186/1471-2334-13-S1-P75

**Published:** 2013-12-16

**Authors:** Nina-Ioana Șincu, Lucia Carmen Chiriac, Brînduşa Ţilea, Erzsebet Iringo Zaharia-Kezdi, Anca Meda Georgescu, Cristina Gârbovan, Andrea Incze, Andreea Bodea, Simona Bățagă

**Affiliations:** 1 Department of Infectious Diseases, University of Medicine and Pharmacy Tîrgu Mureş, Romania; 2 Clinic of Infectious Diseases I, Clinical County Hospital Mureş, Romania; 3 Department of Internal Medicine I, University of Medicine and Pharmacy Tîrgu Mureş, Romania

## Background

*Helicobacter pylori*, responsible for one of the most frequent infections on the globe, was reported by several literature studies to be less frequent among patients infected with human immunodeficiency virus (HIV), especially at low levels of CD4+ T-cells. The purpose of the study was to evaluate the features of *Helicobacter pylori* infection in HIV-positive patients from the Mureş regional center.

## Methods

We performed a retrospective, descriptive study, over a 3-year period (August 2010 – August 2013), on 63 HIV-positive patients with dyspeptic symptoms, tested for *Helicobacter pylori* infection in the Laboratory of Infectious Diseases, Clinical County Hospital Mureş.

## Results

Seventeen (26.98%) HIV-positive patients were co-infected with *Helicobacter pylori*. The average level of CD4+ T-lymphocytes was 292 cells/µL in *Helicobacter pylori*-positive patients and 312 cells/µL in *Helicobacter pylori*-negative ones, which did not result in a statistically significant difference regarding the level of CD4+ T-cells (p=0.8785).

## Conclusion

Our study showed a frequency of *Helicobacter pylori*-HIV co-infection of 26.98% among HIV-positive patients monitored in the regional center Mureş, without statistically significant differences regarding the level of CD4+ T-cells.

